# Down-regulation of miR-361-5p promotes the viability, migration and tube formation of endothelial progenitor cells via targeting FGF1

**DOI:** 10.1042/BSR20200557

**Published:** 2020-10-16

**Authors:** Xiaofeng Yang, Yanli Song, Yuexi Sun, Mengmeng Wang, Yang Xiang

**Affiliations:** Department of Emergency, Tongji Hospital, Tongji University School of Medicine, Shanghai, China

**Keywords:** deep venous thrombosis recanalization, endothelial progenitor cells, Fibroblast Growth Factor 1, miR-361-5p

## Abstract

Transplantion of bone marrow-derived endothelial progenitor cells (EPCs) may be a novel treatment for deep venous thrombosis (DVT). The present study probed into the role of microRNA (miR)-361-5p in EPCs and DVT recanalization. EPCs were isolated from male Sprague–Dawley (SD) rats and identified using confocal microscopy and flow cytometry. The viability, migration and tube formation of EPCs were examined using MTT assay, wound-healing assay and tube formation assay, respectively. Target gene and potential binding sites between miR-361-5p and fibroblast growth factor 1 (FGF1) were predicted by StarBase and confirmed by dual-luciferase reporter assay. Relative expressions of miR-361-5p and FGF1 were detected using quantitative real-time polymerase chain reaction (qRT-PCR) and Western blot as needed. A DVT model in SD rats was established to investigate the role of EPC with miR-361-5p antagomir in DVT by Hematoxylin–Eosin (H&E) staining. EPC was identified as 87.1% positive for cluster of difference (CD)31, 2.17% positive for CD133, 85.6% positive for von Willebrand factor (vWF) and 94.8% positive for vascular endothelial growth factor receptor-2 (VEGFR2). MiR-361-5p antagomir promoted proliferation, migration and tube formation of EPCs and up-regulated FGF1 expression, thereby dissolving thrombus in the vein of DVT rats. FGF1 was the target of miR-361-5p, and overexpressed FGF1 reversed the effects of up-regulating miR-361-5p on suppressing EPCs. Down-regulation of miR-361-5p enhanced thrombus resolution *in vivo* and promoted EPC viability, migration and angiogenesis *in vitro* through targeting FGF1. Therefore, miR-361-5p may be a potential therapeutic target for DVT recanalization.

## Introduction

Deep venous thrombosis (DVT) refers to the formation of a blood clot within a deep vein in contrast with venous thromboembolism, which includes superficial thrombophlebitis and pulmonary embolism [1]. DVT will lead to morbidity and mortality in several conditions [2]. Anti-coagulation is a major therapeutic strategy for DVT, but it cannot dissolve thrombus and restore the function of valves, at the same time, thrombosis may occur with post-thrombotic syndrome (PTS) [3]. Thus, it is of great significance to explore a new therapeutic method for DVT treatment.

Recent discoveries showed that successful DVT-related thrombi resolution plays a key role in DVT treatment [4], however, detailed mechanisms remained obscure. Endothelial progenitor cells (EPCs), derived from bone marrow and resident to tissues, act as precursor for endothelial cells. EPCs have the ability to differentiate into mature endothelial cells and play a major role in vascular integrity maintenance and endothelial damage as well as the resolution of thrombus *in vivo* [5–7]. EPCs can be homed and integrated into the injured blood vessel and thrombus to secrete angiogenesis factors, thus increasing the formation of new blood vessels and improving the resolution of vascular thrombosis when DVT occurs [2]. Therefore, the effective recruitment of EPCs into thrombus may help treat DVT.

As previous study demonstrated, microRNAs (miRNAs, miRs) are involved in the biological functions of EPCs [8]. MiRNAs are a highly conserved family of small non-coding RNAs (ncRNAs) with 19–25 nucleotides in length and regulate the gene expression through the combination with 3′-untranslated region (3′-UTR) of target messenger RNAs (mRNAs) [9]. MiR-361-5p, in particular, has been reported to suppress many human cancers progression, such as hepatocellular carcinoma [10], hemangioma [11] and papillary carcinoma [12]. According to Wang et al.’s study, miR-361-5p suppresses vascular endothelial growth factor (VEGF) expression and EPC activity [13]. We predicted that the target gene of miR-361-5p was fibroblast growth factor 1 (FGF1), which aroused our research interest. FGF1, which plays a part in cell proliferation, migration, invasion and angiogenesis, can promote the viability of EPCs and neovascularization [14,15]. Herein, in our study, the roles of miR-361-5p in the development and progression of DVT were explored through examining their regulatory functions in EPCs based on an animal model, hoping to find a potential therapeutic method for DVT.

## Materials and methods

### EPCs isolation and culture

EPC isolation was conducted following a previous description [16]. Male Sprague–Dawley (SD) rats (3 weeks old, 80–100 g) were purchased from Guangdong Medical Laboratory Animal Center (Foshan, China) and kept in microisolator cages under a 12-hour (h) day/night cycle at 23°C with free access to standard laboratory diet and tap water for 2 weeks before our experiment. Then, after 2 weeks of stabilization, five male SD rats were anesthetized and sacrificed with intraperitoneal injection of ketamine (100 mg/kg) and xylazine (10 mg/kg). Subsequently, bone marrow of the rats was harvested via femurs and tibias. Mononuclear cells were available from density-gradient centrifugation with Ficoll-Paque (GE Healthcare, Piscataway, NJ, U.S.A.). EPCs at the density of 0.8–1.0 × 10^6^ cells/cm^2^ were inoculated to the culture flask and cultured with microvascular endothelial cell growth-2 (EGM-2) medium; catalog number: CC-3125; Lonza, Greenwood, SC, U.S.A.), which contained penicillin-streptomycin (P4333, Sigma-Aldrich, St Louis, MO, U.S.A) at 37°C with 5% CO_2_. Non-adherent cells were washed after the cell culture for 4 days, and the medium was refreshed every 2 days. EPCs in passage 3 were selected for subsequent experiments.

### EPC identification

EPCs were characterized by both confocal microscopy and flow cytometry. For detection by flow cytometry, in detail, on day 7, the cells were first isolated and blocked with 2% FBS at 4°C for 10 min, then washed with phosphate buffered saline (PBS) and separately incubated with primary antibodies as fluorescein isothiocyanate (FITC)-conjugated VEGF receptor-2 (VEGFR-2) antibody (ab184903, Abcam, Cambridge, U.K.), FITC-conjugated von Willebrand factor (vWF) antibody (ab8822, Abcam, U.K.), FITC-conjugated cluster of difference (CD)31 antibody (ab33858, Abcam, U.K.) or CD133 antibody (ab18898, Abcam, U.K.) at 4°C for 30 min. Goat Anti-Rabbit IgG H&L (Alexa Fluor® 488, ab150077, Abcam, U.K.) as the secondary antibody was used for CD133 staining. After the incubation, the cells were washed with PBS containing 0.1% bovine serum albumin (A7030, Sigma–Aldrich, U.S.A.) and then analyzed with fluorescence-activated cell sorting (FACS) in FacsCalibur™ flow cytometer (BD Biosciences, Franklin Lakes, NJ, U.S.A.). For identification under confocal microscopy Cells were incubated with 1,1′-dioctadecyl-3,3,3′,3′-tetramethylindocarbocyanine perchlorate (Dil)-labeled acetylated low-density lipoprotein (Dil-Ac-LDL, BT-902, Bioquote, York, UK) and fluorescein isothiocyanate (FITC)-labeled Ulex europaeus agglutinin 1 (FITC-UEA-1, L32476, Invitrogen, Carlsbad, CA, U.S.A). Double staining of Dil-ac-LDL and FITC-UEA-1 under confocal microscope (Leica Microsystems GmbH, Germany) suggested that the isolated cells were identified as EPCs.

### Cell transfection with agomir and antagomir

The harvested EPCs were cultured in M199 basic medium (M9163, Sigma-Aldrich, U.S.A.) containing 10% FBS (F2442, Sigma-Aldrich, U.S.A.), 1% penicillin-streptomycin in a humidified incubator at 37°C with 5% CO_2_. Then, to overexpress and knock down miR-361-5p in EPCs, miR-361-5p agomir (sequence: 5′-UUAUCAGAAUCUCCAGGGGUAC-3′) and antagomir (sequence: 5′-GUACCCCUGGAGAUUCUGAUAA-3′) were purchased from Gene Pharma (Shanghai, China). After the cells reached 70–80% confluence, miR-361-5p agomir and antagomir at a concentration of 50 nM were mixed with the medium containing Lipofectamine 2000 reagent (11668-500, Invitrogen, U.S.A.) in accordance with the manufacturer’s protocol. For further studies both *in vivo* and *in vitro*, miR-361-5p agomir and antagomir were maintained in EPCs for at least 14 days.

### RNA isolation and quantitative real-time polymerase chain reaction (qRT-PCR)

Total RNA from EPCs was extracted using TRIzol reagent (15596-018, Invitrogen, Madison, MI, U.S.A.) following the protocols of the manufacturer and preserved in a 4°C or −80°C refrigerator. Concentration of total RNA was detected and quantified using a biological spectrometer (Nano Drop 2000, Thermo Fisher, Waltham, MA, U.S.A.). The cDNA was synthesized from 1 μg of total RNA with a First-Strand cDNA Synthesis Kit (E6300L; New England Biolabs, Beijing, China) according to the manufacturer’s instructions. QRT-PCR was conducted with SYBR Premix ExTaq II kit (RR820L, TaKaRa, Shiga, Japan) in Touch Real-Time PCR Detection system (CFX96, Bio-Rad, U.S.A.) under the following conditions: 95°C for 3 min, followed by 55 cycles at 53°C for 1 min, 72°C for 30 s. Primer sequences for this experiment are shown in Table 1. GAPDH (for FGF1) and U6 (for miR-361-5p) were used as internal reference. Relative expressions were quantified by 2^−ΔΔ*C*_T_^ calculation method [17].

**Table 1:** Primers for qRT-PCR

Gene	Primers
*miR-361-5p*	
Forward	5′-AGGGGTACGTCGTATCCAGT-3′
Reverse	5′-GTATCCAGTGCGTGTCGTGG-3′
*FGF1*	
Forward	5′-GCAAGGTTTTGGTGCTTACC-3′
Reverse	5′-TCGATGGTGCGTTCAAGAC-3′
*GAPDH*	
Forward	5′-TGGCCACGCTAATCTGACT-3′
Reverse	5′-GGTAACCAGGCGTCCGATA-3′
*U6*	
Forward	5′-GCACATTCTCCCCAGTTATGA-3′
Reverse	5′-TCACAAATTTGCATGTCATCCT-3′

### MTT assay

MTT assay was performed to measure the EPC viability. In brief, the EPCs (1 × 10^3^ cells/well) were cultured in 96-well plates and 10 μl MTT assay kit (#30006, Biotium, Inc., Fremont, CA, U.S.A.) was added into the wells at 12, 24 and 48 h of the culture. The supernatant was discarded 4 h after incubation at 37°C, and 100 μl dimethyl sulfoxide (DMSO; 472301, Sigma–Aldrich, U.S.A.) was added into the wells to dissolve formazan crystals. The OD values at an absorbance of 490 nm were measured and recorded by a microplate reader (Model 680, Bio-Rad, U.S.A.).

### Wound healing assay

After 48-h cell transfection, the EPCs (1 × 10^4^ cell/ml) were seeded in a 24-well tissue culture plate. The straight wound in the middle of the culture was then created by a sterile pipette tip after the cells reached 100% confluence. After washing the cells by PBS twice to smoothen the edge of scratch and the removal of the floating cells, the EPCs were incubated in an incubator at 37°C with 5% CO_2_. Cell images at 0 and 48 h were captured under an inverted optical microscope (SW380T, Swift Optical Instruments, Schertz, TX, U.S.A.). Cell migration was measured by Image-Pro Plus Analysis software (Version 6.0, Media Cybernetics Company, U.S.A.).

### Tube formation assay

The vascular formation of EPCs was evaluated using tube formation assay and Matrigel plug assay. Pre-heated matrigel (R&D Systems, Minneapolis, MN, U.S.A.) at 4°C overnight was diluted with non-serum medium, layered in 96-well plates and incubated at 37°C for 30 min to allow polymerization. Subsequently, the EPCs (2 × 10^4^ cell/ml) were plated on to the Matrigel layer in EGM™-2 MV medium, and later the capillary-like structures formation was captured using an inverted microscope (IRB20, Microscope World, Carlsbad, CA, U.S.A.) with Tube formation ACAS Image Analysis Software (v.1.0, ibidi GmbH, Gräfelfing, Germany).

### Target gene and dual-luciferase reporter assay

StarBase (http://www.starbase.sysu.edu.cn) predicted that the target gene of miR-361-5p was *FGF1*, which aroused our research interest. Previous study found that miR-361-5p plays an important role in cell proliferation and invasion [18]. FGF1 has also been found to promote the activity, proliferation, angiogenesis and anti-apoptosis of EPCs [15]. The predicted targeted relationship was subsequently confirmed by dual-luciferase reporter assay.

PMIR-REPORT Luciferase vector (catalog number: AM5795; Thermo Fisher Scientific, U.S.A.) containing the sequences of wildtype or mutated FGF1 3′-UTR was cloned into the pMirGLO reporter vector (Promega, Madison, WI, U.S.A.) to form FGF1-WT and FGF1-MUT. A total of 1 × 10^4^ cells/ml (EPCs) were then co-transfected with FGF1-WT and FGF1-MUT, miR-361-5p agomir and miR-NC-agomir by Lipofectamine 2000 Transfection reagent (Thermo Fisher Scientific, U.S.A.) at 37°C. *Renilla* reporter gene in the luciferase reporter vector was used as an internal control. Then cells were harvested 48 h after the transfection for luciferase detection in dual-luciferase reporter assay system (E1910; Promega, Madison, WI, U.S.A.) following the producer’s protocols. The firefly luciferase activity was normalized to that of *Renilla* luciferase activity.

### Western blot

FGF1 protein expressions in P3 EPCs were measured using Western blot as previously described [19]. In brief, the proteins were lysed and extracted from EPCs with RIPA buffer (catalog number: P0013C; Beyotime, Shanghai, China), and then bicinchoninic acid (BCA) protein kit (catalog number: B9643; Sigma–Aldrich, U.S.A.) was used to measure protein concentration. Thirty microgram lysates of sample protein were electrophoresed by 12% sodium dodecyl sulfate/polyacrylamide gel electrophoresis (SDS/PAGE; P0012A; Beyotime, China), followed by transferring into polyvinylidene fluoride (PVDF; FFP28; Beyotime, China) membrane. The film was blocked with 5% nonfat milk for 2 h at room temperature and incubated in the following primary antibodies: anti-FGF1 antibody (ab179455, rabbit, 1:1000, Abcam, Cambridge, U.K.) and anti-GAPDH antibody (ab181602, rabbit, 1:10000, Abcam, U.K.) at 4°C overnight. GAPDH was used for internal reference. Secondary horseradish peroxidase (HRP)-combined antibody goat anti-rabbit IgG H&L (goat, 1:2000, ab205718, Abcam, U.K.) was used to further incubate with the film for 1 h at room temperature and washed with Tris-buffer saline tween (TBST) for three times. Protein band was collected from the samples and analyzed in an enhanced chemiluminescence (ECL) kit (Millipore, Billerica, MA, U.S.A.). The gray values of the strips were further gathered and calculated by ImageJ (version 5.0; Bio-Rad, U.S.A.).

### Construction of animal model

SD rats (10 weeks old, 280–300 g) regardless of gender were obtained from Guangdong Medical Laboratory Animal Center (Foshan, Guangdong, China), and were then kept in specifically made pathogen-free animal rooms. For this, rat model construction was well described in previous studies [2,16]. In brief, the rats were anesthetized by intraperitoneal 7% pentobarbital injection and underwent midline laparotomy to dissect inferior vena cava (IVC) from aorta. IVC was subsequently ligated just below the upper renal vein using 7-0 Prolene sutures, meanwhile, the posterior venous branches were tightened. Then, confluence in iliac vein was discontinued using a pair of vascular clip for 15 min. After that, the incision was closed and the rats were allowed to recover after the surgery. The rats in Sham group received a dissection of IVC but without ligation.

Three days after the construction of animal model, the SD rats were divided into four groups at random (total number = 40; *n*=10 for each group): (A) Sham group received 2 ml EGM™-2 MV medium (Lonza, U.S.A.) injection with IVC exposure, (B) Model group received 2 ml EGM™-2 MV medium injection after model construction, (C) EPCs group received the injection of 1 × 10^6^ EPCs containing miR-NC carriers via tail vein injection, (D) EPC+miR-361-5p antagomir group received injection of 1 × 10^6^ EPCs via tail vein injection after transfection with miR-361-5p antagomir.

### Histopathologic examination with Hematoxylin–Eosin (H&E) staining

The rats were anesthetized and sacrificed with intraperitoneal injection of ketamine (100 mg/kg) and xylazine (10 mg/kg) 7 days after the injection. Segments of IVC containing the thrombus were harvested with caution and fixed in 4% paraformaldehyde, subsequently embedded in dissolved paraffin. Excess blood on thrombi was removed using filter paper. Specimens were finally stained with Hematoxylin–Eosin (H&E) and analyzed with a light inverted microscope (CKX53; Olympus, Tokyo, Japan) in the dark.

### Statistical analysis

In our study, all the experiments were independently performed more than three times. The experimental data were expressed as mean ± standard deviation. Statistical analysis was performed with SPSS 21.0 software (IBM Corporation, Armonk, NY, U.S.A.). Normal distribution and variance homogeneity were tested for all the data. Comparison of differences between multiple groups was determined by one-way ANOVA. Comparison of differences between two groups was determined by Student’s *t* test. *P*-value <0.05 was considered as statistically significant.

## Results

### Culture and identification of EPCs

**Figure 1:**
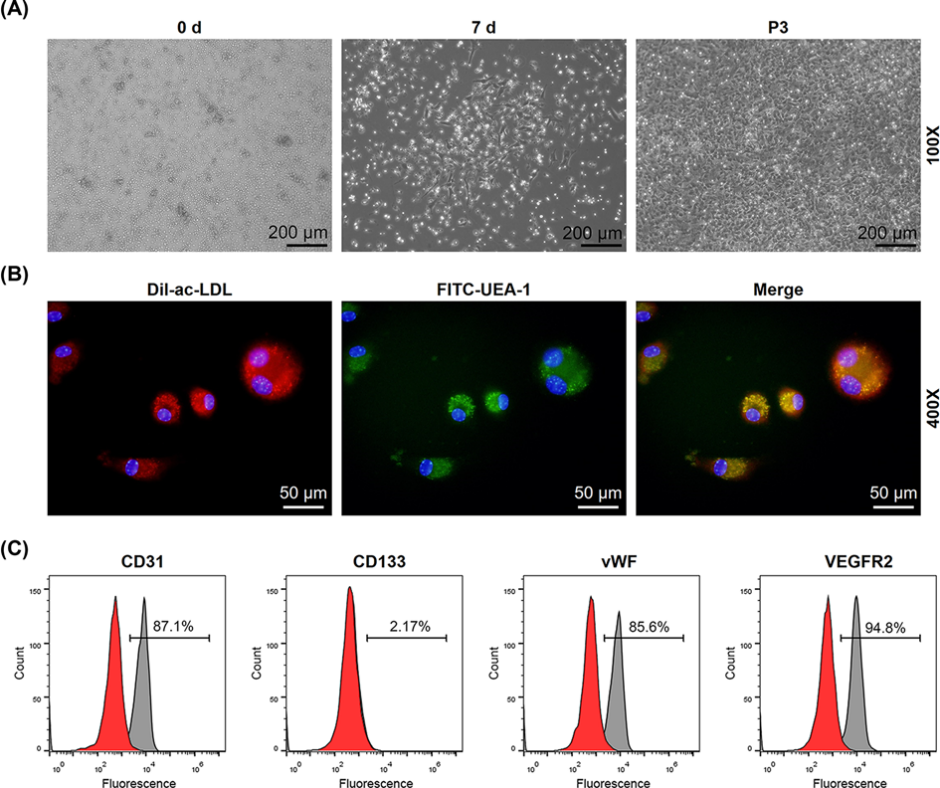
Characterization of EPCs (**A**) The changes in morphology of EPCs (days 0 and 7, and passage 3) were observed using an inverted optical microscope, under 100× magnification. (**B**) Staining of Dil-Ac-LDL, FITC-UEA-1 and merged image of double staining of Dil-Ac-LDL and FITC-UEA-1 using confocal microscope, under 400× magnification. (**C**) Flow cytometry analysis showed the expressions of CD31, CD133, vWF and VEGFR2 on EPCs.

In line with previous studies, changes in the morphology and numbers of EPCs were observed under an inverted optical microscope. Shortly after the isolation, colony of Peripheral blood mononuclear cells (PBMCs) exhibited a round morphology and suspended in the medium (Figure 1A). Then, 7 days after the culture, an elongated spindle-shaped morphology and formed central cluster was observed (Figure 1A). Also, PBMCs began to merge in passage 3 (Figure 1A). The isolated PBMCs were identified by confocal microscopy, and the double staining with functional marker FITC-UEA-I and Dil-Ac-LDL suggested that the isolated PBMCs were EPCs (Figure 1B). EPCs were further characterized by flow cytometry. CD31, CD133, vWF and VEGFR2 were markers of EPCs [20], and therefore their expressions were measured using flow cytometry in order to confirm the identity of EPCs. In Figure 1C, the results from flow cytometry showed that P10 cells were 87.1% positive for CD31, 2.17% positive for CD133, 85.6% positive for vWF and 94.8% positive for VEGFR2, suggesting that the isolated mononuclear cells were EPCs.

### MiR-361-5p antagomir reduced miR-361-5p expression yet promoted cell viability, migration and tube formation of EPCs

**Figure 2:**
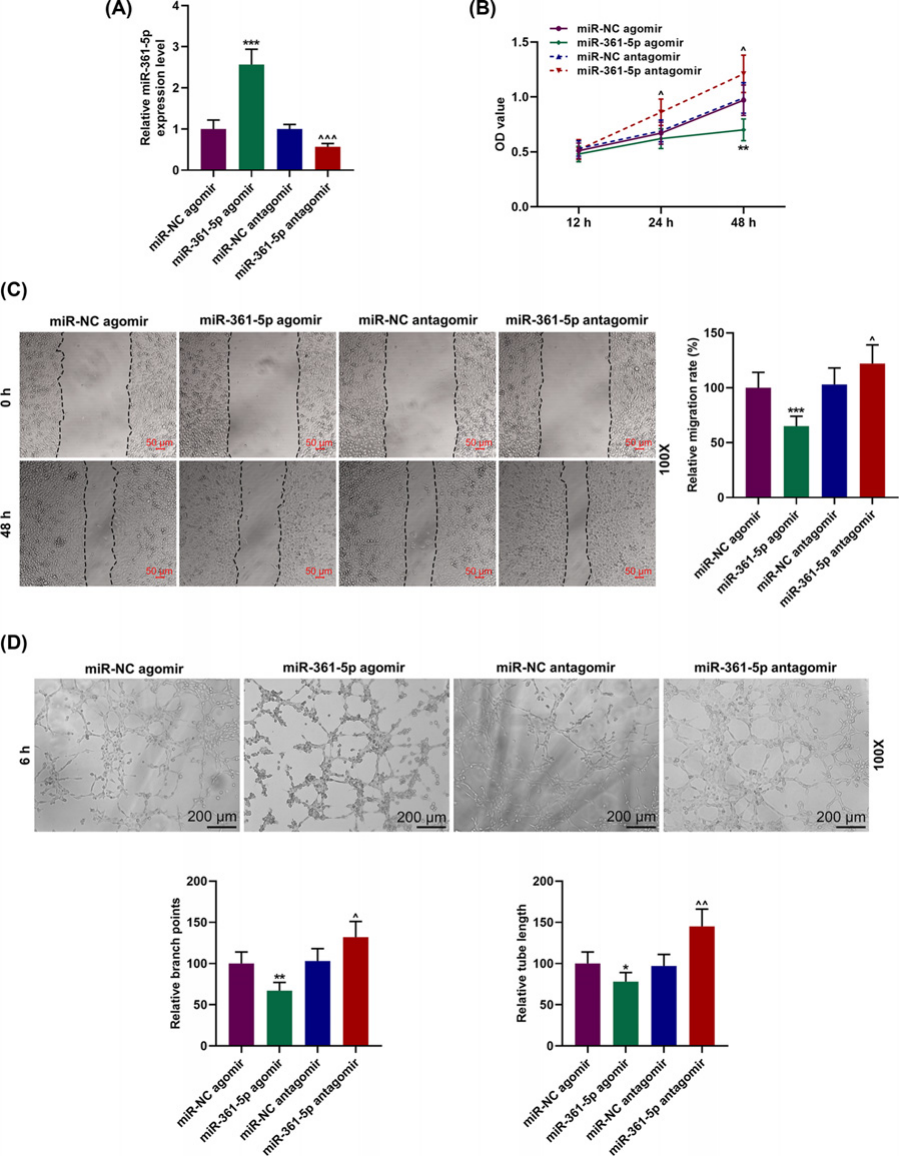
Role of miR-361-5p on the proliferation, migration and tube formation capability of EPCs (**A**) EPCs were transfected with miR-361-5p agomir or antagomir, and their respective negative control groups (miR-NC agomir; miR-NC antagomir) were established. The transfection rates were measured by qRT-PCR. (**B**) The viability of EPCs which have been transfected with miR-361-5p agomir or miR-361-5p antagomir at 12, 24 and 48 h were detected by MTT assay. (**C**) The relative cell migration rate of EPCs was measured by wound-healing assay at 0 and 48 h, under 100× magnification. (**D**) The relative branch points and relative tube length of EPCs were detected using tube formation assay at 6 h, under 100× magnification. *P<0.05, **P<0.01, ***P<0.001, vs. miR-NC agomir; ^∧^*P*<0.05, ^∧∧^*P*<0.01, ^∧∧∧^*P*<0.001, vs. miR-NC antagomir. All experiments have been performed in triplicate.

The EPCs were transfected with miR-361-5p agomir or antagomir for examining the role of miR-361-5p in the functions of EPCs; accordingly, their respective negative control groups (miR-NC agomir; miR-NC antagomir) were also established. We firstly detected and quantified miR-361-5p transfection rate with qRT-PCR, and found that miR-361-5p expression in miR-361-5p agomir group was evidently up-regulated, while that in miR-361-5p antagomir group was down-regulated, as compared with their respective negative control groups (Figure 2A, *P*<0.001). This indicated that miR-361-5p agomir could increase miR-361-5p expression in EPCs while miR-361-5p antagomir had the opposite effect.

To further uncover the effects of miR-361-5p on EPC functions, we measured the viability, migration and tube formation of EPCs after the transfection with miR-361-5p agomir or antagomir. MTT assay showed that the viability of EPCs at 24 and 48 h was reduced in miR-361-5p agomir group (Figure 2B, *P*<0.01), while that in miR-361-5p antagomir group showed an opposite result at 24 and 48 h (Figure 2B, *P*<0.05), indicating that down-regulating miR-361-5p could promote the cell viability. Then the migration of EPCs was determined using wound-healing assay. The data from the experiments revealed that relative migration rate of EPCs in miR-361-5p agomir group was decreased (Figure 2C, *P*<0.001), while that in miR-361-5p antagomir group was increased (Figure 2C, *P*<0.05), suggesting that down-regulating miR-361-5p expression promoted the migration of EPCs. Finally, the EPC tube formation was detected with tube formation assay, and it has been observed that both branch points and relative tube length in miR-361-5p agomir group were reduced (Figure 2D, *P*<0.01), while that in miR-361-5p antagomir group was increased (Figure 2D, *P*<0.01), suggesting that the EPC tube formation could be enhanced by down-regulating miR-361-5p.

### FGF1 was the target of miR-361-5p and overexpressed FGF1 reversed the effects of miR-361-5p agomir on FGF1 expression

**Figure 3:**
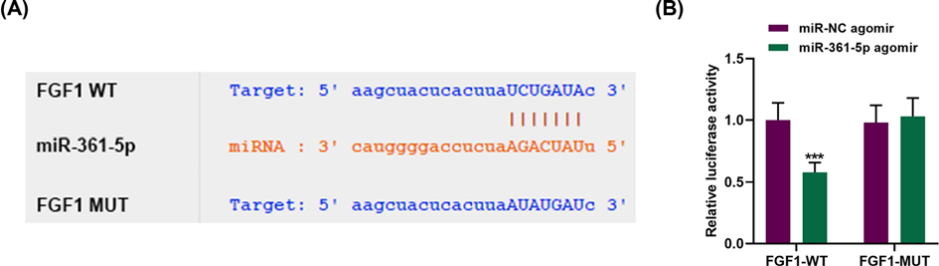
FGF1 was the target of miR-361-5p (**A**) Sequences of FGF1-WT (top), miR-361-5p (middle) and FGF-1-MUT (below) were listed. (**B**) Dual-luciferase reporter assay showed that FGF1 was the target of miR-361-5p. ***P<0.001, vs. miR-NC agomir.

MiRNAs combine with 3′-UTR of target mRNAs to regulate gene expressions [9]. By applying StarBase, we successfully found that FGF1 might be a possible target of miR-361-5p, because it contained miR-361-5p binding sites at 3′-UTR (Figure 3A). To further confirm that miR-361-5p could bind with FGF1, we built a luciferase reporter vector containing 3′-UTR. For the assay, the results demonstrated that the relative luciferase activity in FGF1-WT group was reduced in the presence of miR-361-5p agomir (Figure 3B, *P*<0.001). However, no significant difference was detected in luciferase activity of miR-361-5p agomir in FGF1-MUT group (Figure 3B). These results suggested that FGF1 was the target of miR-361-5p.

**Figure 4:**
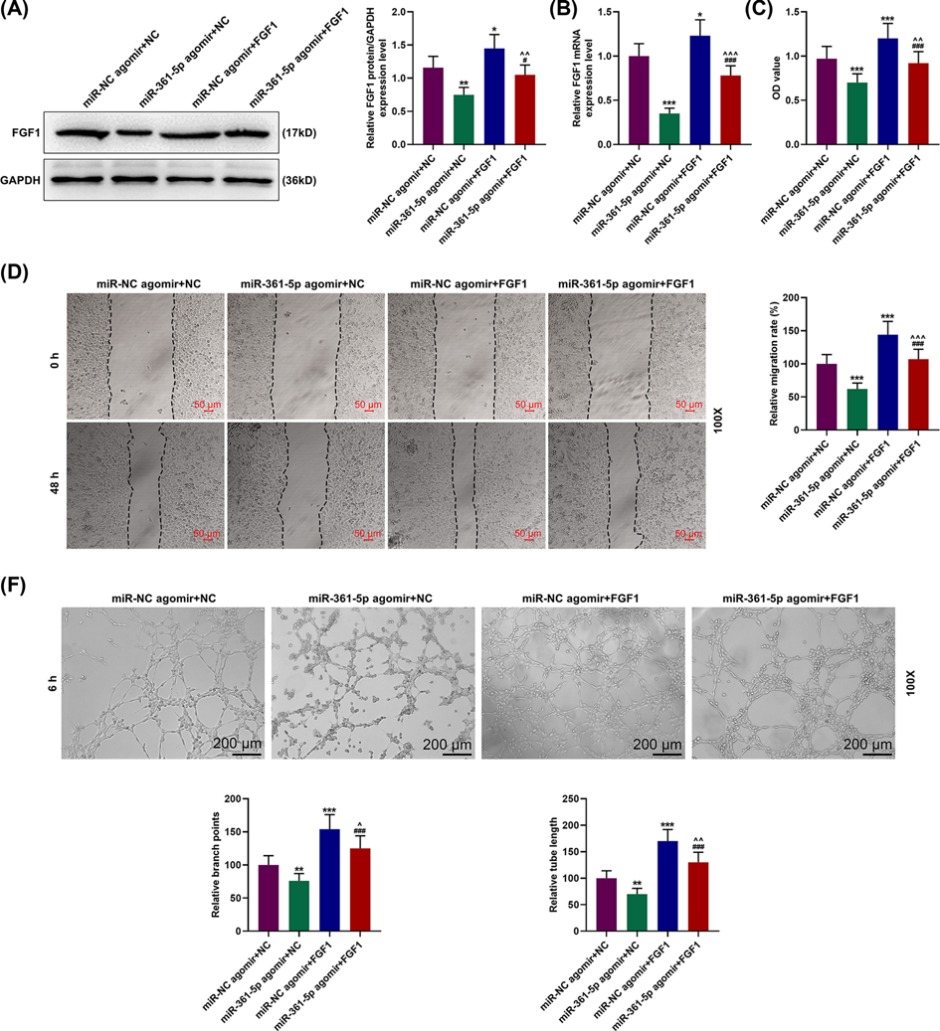
MiR-361-5p agomir inhibited the EPCs viability, migration and tube formation capability through regulating FGF1 (**A**) Relative FGF1 protein expressions were measured by Western blot. GAPDH was the internal reference. (**B**) Relative FGF1 mRNA expressions were measured by qRT-PCR.GAPDH was the internal reference. (**C**) EPCs viability were detected using MTT assay. (**D**) Relative migration rates of EPCs were observed using wound-healing assay at 0 and 48 h, under 100× magnification. (**E**) Relative branch points and tube length of EPCs were detected using tube formation assay at 6 h, under 100× magnification. *P<0.05, **P<0.01, ***P<0.001, vs. miR-NC agomir+NC; ^#^*P*<0.05, ^###^*P*<0.001, vs. miR-NC agomir+FGF1; ^∧^*P*<0.05, ^∧∧^*P*<0.01, ^∧∧∧^*P*<0.001, vs. miR-361-5p agomir+NC. All experiments have been performed in triplicate and experimental data were expressed as mean ± standard deviation (SD).

To further determine the effects of miR-361-5p on FGF1 expression in EPCs, the transfection of miR-361-5p agomir and antagomir was carried out for determining the FGF1 expressions. As illustrated in Figure 4A,B, relative FGF1 expressions were down-regulated after miR-361-5p agomir transfection (*P*<0.001), but up-regulated in after FGF1 overexpression (*P*<0.05). Furthermore, overexpressed FGF1 reversed the effects of miR-361-5p on FGF1 protein and mRNA expressions on EPCs (Figure 4A,B, *P*<0.001).

### Overexpression of FGF1 reversed the inhibitory effects of miR-361-5p agomir on the viability, migration and tube formation of EPCs

To uncover the effects of miR-361-5p and FGF1 on the EPC viability, migration and tube formation, EPCs were transfected with miR-361-5p agomir or antagomir. MTT assay showed that the EPC viability was reduced after miR-361-5p agomir was transfected into the cells, while overexpressed FGF1 showed an opposite effect (Figure 4C, *P*<0.001). In addition, overexpressed FGF1 reversed the effects of miR-361-5p agomir on the EPC viability (Figure 4C, *P*<0.01). In wound-healing assay, relative migration of EPCs in miR-361-5p agomir+NC group was reduced, whereas overexpression of FGF1 led to an opposite result (Figure 4D, *P*<0.001). Furthermore, overexpressing FGF1 in EPCs reversed the effects of miR-361-5p agomir on the cell migration (Figure 4D, *P*<0.001). Moreover, from tube formation assay, it could be observed that the relative branch points and tube length of the EPCs were reduced following upregulating miR-361-5p (Figure 4E, *P*<0.01), while overexpressed FGF1 resulted in an opposite effect (Figure 4E, *P*<0.001), and overexpression of FGF1 in the EPCs reversed the effects of miR-361-5p agomir (Figure 4E, *P*<0.01).

### EPCs with miR-361-5p antagomir showed promotion on thrombus resolution in the vein

**Figure 5:**
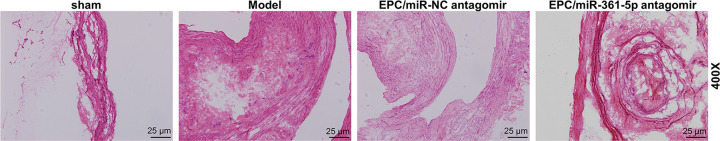
EPCs with MiR-361-5p antagomir could promote thrombus resolution in the vein After Hematoxylin&Eosin staining and miR-361-5p antagomir transfection, histopathological observation on venous thrombi of rats in Sham group, Model (DVT) group, EPC/miR-NC antagomir group and EPC/miR-361-5p antagomir group was performed under an inverted microscope under 400× magnification on day 7 after animal model was established (*n*=10 for each group).

As shown in Figure 5, H&E staining showed a normal vein in Sham group, while in Model group, nucleated cells (monocytes, endothelial cells and neutrophil granulocytes) were found entering the thrombus perimeter on day 7. Moreover, the red blood cells, platelets and fibrin were dried red in the center of thrombus in Model group. Besides, in EPC/miR-NC antagomir group, more nucleated cells and channels with reduced thrombus were detected on day 7 compared with Model group. In addition, in EPC/miR-361-5p antagomir group, more nucleated cells were found entering the thrombus. For EPC/miR-361-5p antagomir group, we observed small fracture in the perimeter of thrombus and the formation of tube structure and red blood cells. Collectively, the experimental results suggested that EPCs with miR-361-5p antagomir promoted thrombus resolution in the vein.

## Discussion

Thrombosis, which referred to the blood clot formation inside blood vessels, could obstruct blood flow in the circulatory system [16]. Endothelial cells at normal state express the molecules with anticoagulant effect and inhibit the formation of fibrin [21]. Moreover, as endothelial cells may induce tissue repair and tube formation [22], it now plays an important role in thrombosis prevention and treatment. EPCs function as precursor cells for mature endothelial cells, and its potential to differentiate into all capillary niches allows it to contribute to vascularizing engineered tissues [23]. Many researches uncovered the relation between proliferation, migration and tube formation of EPCs and DVT recanalization. Mo *et al*. demonstrated that down-regulation on miR-195 could regulate the proliferation, migration, angiogenesis and autography of EPCs by targeting GABA type A receptor-associated protein-like 1 (GABARAPL1), and Li *et al*. discovered that miR-3120 was implicated in the mechanisms via which long non-coding RNA Wilms Tumor 1 Associated Protein Pseudogene 1 (LncRNA WTAPP1) promoted EPCs migration and angiogenesis [24,25]. In our study, consistent with previous discoveries, we found that EPCs transfected with miR-361-5p antagomir could partially promote thrombus resolution in vein.

Recently, the functions of miRNAs in the regulation of vascular development, homeostasis and differentiation have been widely explored both at home and abroad [26,27]. MiRNAs also affect EPC function in angiogenesis [28]. MiR-361-5p, in particular, has been found overexpressed in vascular cells, including EPCs, to inhibit their activities [13]. Wang *et al*. demonstrated that miR-361-5p could suppress EPCs activities via targeting VEGF in patients with coronary artery diseases [29]. In our present study, we conducted a series of studies on the biological behaviors of EPCs at the cellular level, and found that after miR-361-5p antagomir was transfected into EPCs, the viability, migration and tube formation of EPCs were promoted, suggesting that down-regulating miR-361-5p may have promoting effects on EPCs, which was consistent with previous studies [29]. However, upregulating miR-361-5p significantly reduced the viability, migration and angiogenesis of EPCs. Such a result encouraged us to further study its mechanism of action. Using the starBase site, we predicted the target genes and potential binding sites of miR-361-5p. FGF1, which was predicted as a target for miR-361-5p in the present study, has been found to promote activity, proliferation and angiogenesis of EPCs [15]. Moreover, we also confirmed the targeting relationship between miR-361-5p and FGF1 by dual-luciferase reporter gene assay.

FGF1 is a member of FGF family and a growth factor involved in cell proliferation, migration, invasion and angiogenesis [14]. Up-regulating FGF1 expression modulates to ameliorate atherosclerosis [30]. FGF1 induced by ERK1/2 signaling could reciprocally regulate proliferation and smooth muscle cell differentiation of ligament-derived EPC-like cells [31]. In addition, adipose-derived mesenchymal stem cells (AD-MSCs) transfected with FGF1 has been found to promote angiogenic proliferation [32]. However, the relation of FGF1 with miR-361-5p was hardly discussed. In our studies, according to the results of bioinformatics analysis, a binding site between FGF1 and miR-361-5p was identified, suggesting that FGF1 was the target of miR-361-5p. Then we discovered that down-regulation of miR-361-5p expression could promote FGF1 expression and FGF1 overexpression reversed the effects of up-regulating miR-361-5p on inhibiting the viability, migration and tube formation of EPCs.

MiRNAs also participate in promoting DVT recanalization and resolution [28], but the effects of miR-361-5p in promoting DVT recanalization has not been examined yet. The results of *in vitro* experiments showed that miR-361-5p regulated the proliferation, migration and angiogenesis of EPCs through FGF1. Next, we carried out *in vivo* experiments to further explore the effect of miR-361-5p on venous thrombosis. In our studies, we found that miR-361-5p played an important role in DVT recanalization. Moreover, histopathological observation showed that EPCs transfected with miR-361-5p antagomir promoted DVT recanalization, indicating that down-regulating miR-361-5p expression in EPCs promoted thrombus resolution in vein, and that miR-361-5p could be a potential biomarker for DVT treatment.

Our study has some limitations that should be noted, with limited *in vivo* studies, the mechanisms of action of miR-361-5p on EPCs and DVT recanalization have not been fully elucidated, and this will be addressed in our future studies. The present study did not identify the aging of cells, which is also one of the limitations.

In conclusion, our studies revealed a novel role of miR-361-5p in DVT recanalization based on a rat model *in vivo*, and we also discovered that down-regulation of miR-361-5p expression played a vital role in promoting the viability, migration and tube formation of EPCs by targeting FGF1. Therefore, down-regulation of miR-361-5p could serve as a potential therapeutic strategy for DVT diagnosis and prognosis in clinical practice.

## Abbreviations

AD-MSCs: adipose-derived mesenchymal stem cells
CD: cluster of difference
Dil: 1,1′-dioctadecyl-3,3,3′,3′-tetramethylindocarbocyanine perchlorate
Dil-Ac-LDL: Dil-labeled acetylated low-density lipoprotein
DVT: deep venous thrombosis
EGM-2: endothelial cell growth-2
EPC: endothelial progenitor cell
FGF1: fibroblast growth factor 1
FITC: fluorescein isothiocyanate
FITC-UEA1: fluorescein isothiocyanate-labeled Ulex europaeusagglutinin-1
GAPDH: Glyceraldehyde-3-phosphate
HRP: horseradish peroxidase
H&E: Hematoxylin–Eosin
IVC: inferior vena cava
miRNA: microRNA
mRNA: messenger RNA
MTT: 3- (4,5-dimethylthiazol-2-yl)-2,5-diphenyltetrazolium bromide
OD: optical density
PBMC: Peripheral blood mononuclear cells
PBS: phosphate buffered saline
SD: Sprague–Dawley
VEGF: vascular endothelial growth factor
VEGFR2: vascular endothelial growth factor receptor-2
vWF: von Willebrand factor
3′-UTR: 3′-untranslated region
